# On the occurrence of egg masses of the diamond-shaped squid *Thysanoteuthis rhombus* Troschel, 1857 in the subtropical eastern Atlantic (Canary Islands). A potential commercial species?

**DOI:** 10.3897/zookeys.222.2835

**Published:** 2012-09-21

**Authors:** Alejandro Escánez Pérez, Rodrigo Riera Elena, Ángel Francisco González González, Ángel Guerra Sierra

**Affiliations:** 1 BIOECOMAC, Departamento de Biología Animal (Ciencias Marinas), Universidad de La Laguna, Avenida Astrofísico Francisco Sánchez s/n, 38206, La Laguna, Tenerife, Spain; 2 Centro de Investigaciones medioambientales del Atlántico (CIMA SL), Arzobispo Elías Yanes, 44, 38206 La Laguna, Tenerife, Canary Islands, Spain; 3 Instituto de Investigaciones Marinas (IIM-CSIC), Eduardo Cabello, 6, 36208 Vigo, Spain

**Keywords:** *Thysanoteuthis rhombus*, cephalopods, spawning, egg masses, Subtropical Eastern Atlantic, Canary Islands

## Abstract

Data on opportunistic sightings of diamond-shaped squid *Thysanoteuthis rhombus* egg masses in the Canary Islands (Atlantic Ocean) are presented. A total of 16 egg masses of this species were recorded and photographed from 2000 to 2010 around the western islands of the archipelago (El Hierro, Tenerife and La Gomera). These data reveal the existence of an important spawning area for diamond-shaped squid around the Canary Islands, in subtropical east Atlantic waters. We provide preliminary data for the potential development of an artisanal fishery focused on this species, and a discussion on its potential impacts on the marine ecosystem.

## Introduction

The large oceanic diamond-shaped squid *Thysanoteuthis rhombus* Troschel, 1857 is the only species of the family Thysanoteuthidae. The maximum mantle length of this species of both sexes is the same, 100 cm, possibly 130 cm, and the maximum body weight known is 24 to 30 kg, probably more (Roper and Jerez 2010). It is widely distributed in tropical and subtropical waters and has a diurnal behaviour in the mesopelagic layer migrating to the epipelagic zone during the night for feeding and reproductive purposes. The species life span is about one year and males and females mature at age 6 to 8 months, when the mantle length exceeds 250 mm in males and 500 mm in females (Nigmatullin et al. 1995; Roper and Jerez 2010). *Thysanoteuthis rhombus* egg masses are cylindrical, large, gelatinous and planktonic, floating in the sea-surface of tropical and subtropical oceans, their lengths varying from 0.6 to 1,8 m with diameters from 110 to 300 mm. These masses contain from 35,000 to 75,000 eggs, arranged in two rows forming a spiral with two blunt ends (Nigmatullin et al. 1995). This characteristic morphology has led these spawns to be mistakenly identified as pyrosomes or gelatinous plankton species (Berrill 1966).

To date, few egg masses of *Thysanoteuthis rhombus* have been recorded worldwide, representing only 29 records in the literature. In the Pacific Ocean, egg masses have been observed in the Sea of Japan, Okinawa Islands, and coast of Honshu, Bonin Islands, Izu Islands and Sulawesi (Indonesia) (Misaki and Okutani 1976; Suzuki et al. 1979; Billings et al. 2000; Miyahara et al. 2006). Between 1995 and 2000 four egg masses were found in the western Mediterranean Sea and four other spawns in the Canary Islands (Guerra and Rocha 1997; Guerra et al. 2002). These are the first records of egg masses in the geographic region; however, adult catches have been recorded worldwide (Pulido-López and López-Pinto 2002; Ikeda et al. 2003; Marcic et al. 2008; Bello 2009; Salvat-Torres et al. 2009).

## Material and methods

**Figure 1.**
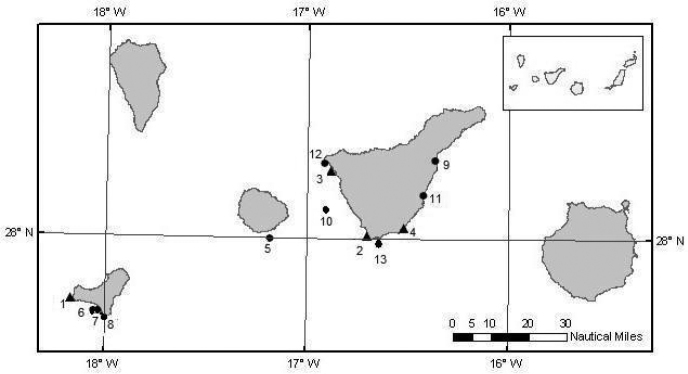
Distribution of egg masses of *Thysanoteuthis rhombus* in Canary Islands. Triangles: literature records. Circles: new data (numbers refer to descriptions in Table 1).

This study is based on a collection of opportunistic sightings of *Thysanoteuthis rhombus* egg masses around the Canary Islands (Fig. 1). The information was compiled from various sources, including sightings by local dive clubs, sightings by researchers during whale watching surveys and literature data. All sighters provided pictures, which allowed accurate identifications. In addition, data on the geographical location of sightings, date, eggs colour and sea surface temperature during the sighting were recorded (Table 1).

**Table 1.** Data on the geographical location of sightings, date, color of eggs and sea surface temperature during the sighting. TF: Tenerife; LG: La Gomera; EH: El Hierro; Temp.: Sea surface temperature.

**N° id.**	**Date**	**Lat., Long.**	**Island**	**N° egg masses**	**Temp. (°C)**	**Color of eggs**	**Locality**	**Authority**
1	October 2000	27°43'N, 18°9.5'W	EH	1	-	no data	Punta Orchilla	Guerra et al. 2002
2	October 2000	28°01'N, 16°42'W	TF	1	-	no data	Punta Rasca	Guerra et al. 2002
3	October 2000	28°18'N, 16°53'W	TF	1	-	no data	Punta Vizcaíno	Guerra et al. 2002
4	October 2000	28°17'N, 16°31'W	TF	1	-	no data	Montaña Pelada	Guerra et al. 2002
5	May 2006	28°01'N, 17°11'W	LG	1	22.8	light pink	Playa Santiago	Herein
6	May 2007	27°40'N, 18°03'W	EH	1	20.0	white	Mar de las Calmas	Herein
7	May 2007	27°40'N, 18°02'W	EH	1	20.0	white	Mar de las Calmas	Herein
8	May 2008	27°38'N, 18°01'W	EH	1	20.5	light pink	Mar de las Calmas	Herein
9	July 2010	28°21'N, 16°22'W	TF	1	22.1	light pink	Candelaria	Herein
10	July 2010	28°10'N, 16°57'W	TF	3	22.1	white	Canal TF-LG	Herein
11	July 2010	28°11'N, 16°25'W	TF	1	22.1	light pink	Las Eras	Herein
12	August 2010	28°20'N, 16°55'W	TF	2	23.0	white	Punta Teno	Herein
13	October 2010	28°02'N, 16°32'W	TF	1	23.7	red	El Médano	Herein

## Results

**Figure 2.**
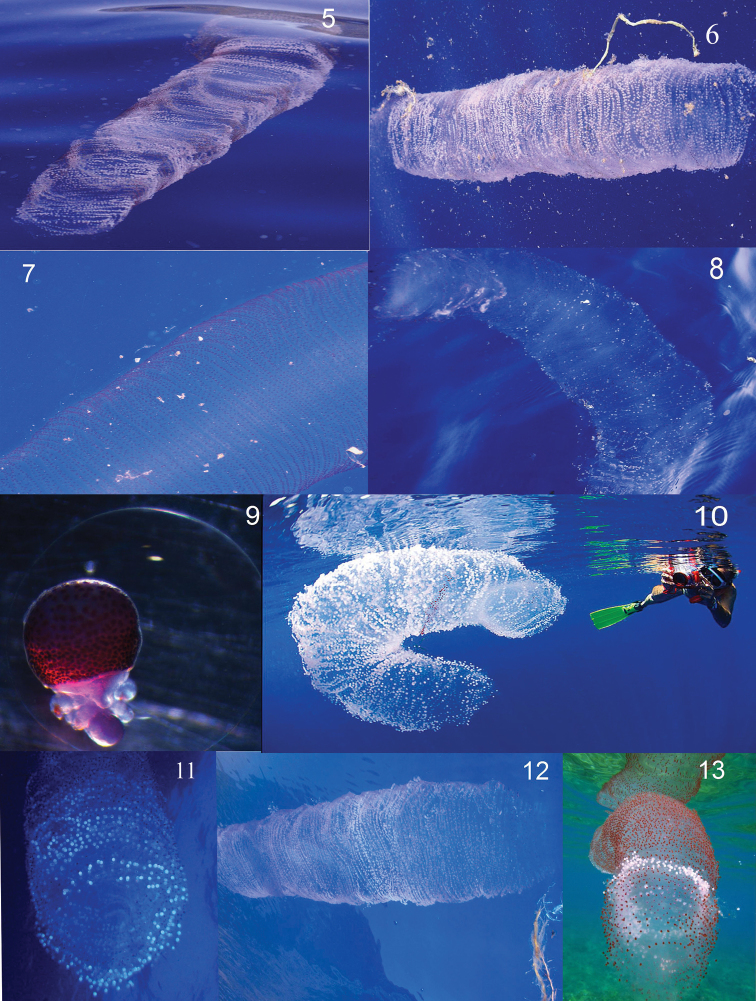
Some *Thysanoteuthis rhombus* egg masses recorded (numbers refer to descriptions in Table 1 and Figure 1).

A total of 16 egg masses were recorded between 2000 and 2010 (Fig. 2). Regardless of the year, egg masses were recorded in summer and early autumn months. The months with the highest number of sightings were May and October. The islands with the highest number of egg masses were Tenerife (11) and El Hierro (5). The presence of egg masses coincided in time with the warmest period of the waters in the Canary Islands, which extends from May to October. Sea surface temperature reached 20°C in June and raised to 24°C in September, decreasing from November to early May below 20°C (18°C in January-February). Thus, egg masses were not recorded in cold waters period. These data seem to reveal the importance of the Canary Islands as a spawning area for diamond-shaped squid in the subtropical eastern Atlantic.

## Discussion

*Thysanoteuthis rhombus* spawns throughout the year in tropical waters, but spawning in subtropical waters is restricted to warm periods (summer and early autumn) and areas with strong warm currents, such as Agulhas and Kuroshio (Nigmatullin et al. 1995). Our observations support this pattern, since the Canary Islands are characterized by intense mesoscale oceanographic structures, such as eddies and warm wakes (Barton et al. 1998).

Adult catches in Canarian waters by local artisanal fishermen are incidental. The species is generally a bycatch of fisheries targeting the ommastrephid squids *Todarodes sagittatus* and/or *Ommastrephes bartramii*. Nevertheless, though *Thysanoteuthis rhombus* catches are currently scarce, it has been considered as a target species with commercial interest in the Canary Islands (Báez and Marrero 2007). In spite of relative frequency of *Thysanoteuthis rhombus* in the catches of that small-scale fishery the presence of mature females in the study area is first recorded herein. This suggests that the Canary Islands are a spawning area for the species. The hypothesis that pelagic egg masses might have been carried out into local waters following spawning elsewhere seems improbable since water temperature from the Canary current and saharian upwelling are colder than coastal Canarian waters.

Moreover, eggs masses found in the Canary Islands varied from white to red. White colour indicates that most eggs have been hatched and red colour is typical of an advanced embryonic stage. Thus, egg masses of different development rates have been recorded in the Canary Islands indicating that this species breeds in the area.

One reason that could account for the low catch of *Thysanoteuthis rhombus* is the gear and the fishing technique used by local fishermen. Gear is deployed by night with a hand-jigging system using light-traps from a small boat, catching the squid on the surface layers about 20 m deep. Another reason that could account for that low catch rate could be the seasonality of fishery targeting ommastrephid squid species, which is restricted to the period between June and August.

In other geographical regions where egg masses of *Thysanoteuthis rhombus* have been recorded, the species supports an important commercial fishery. Thus, in the Sea of Japan (Hyogo Prefecture, Honshu Islands) the fishery has developed since the 1960s, with catches increased annually up to 6,000 metric tons in 2001 (Bower and Miyahara 2005; Takeda and Tanda 1998). The rapid development of this fishery was possible due to the innovation of the fishing gear used by local fishermen. Fishing gear such as the free-floating “*Taru-nagashi*” and “*Hata-nagashi*” were designed specifically for *Thysanoteuthis rhombus*. This gear is deployed primarily in the daytime with 500 meter long free-floating droplines, each made of 2 mm stainless steel multi-strand wire and equipped with a flagpole and a pressure float at one end and three large squid jigs at the other end. Squids are attracted to the gear by a pressure-resistant light snapped onto the mainline, above the squid jigs. This fishing method was widely introduced to other areas of Japan including Okinawa Islands and Ogasawara archipelago (Bonin Islands) (Bower and Miyahara 2005). The search for new fishing grounds of this species has spread beyond the Japanese border. A pilot study carried out in 2004 in Jamaican waters by local institutions under the supervision of the Japan International Cooperation Agency (JICA), found areas with high potential for fishery of this species. The method used to locate these areas in this Japanese pilot study was called the “Egg Trace Method”. In Jamaican waters (Aiken et al. 2007), following the sightings of *Thysanoteuthis rhombus* eggs masses by local fishermen, the fishing gear *“Taru- nagashi”* was deployed in the areas with highest sightings, and the species was successfully captured for the first time.

As mentioned above, *Thysanoteuthis rhombus* is a target species (Bower and Miyahara 2005) and common in fish markets throughout Japan (Omoto et al. 1998). In Okinawa this species occurs more frequently between 400–600 m depth during the day and 50–140 m depth at night (Kanashiro 2001). However, it occurs mainly from 75 to 100 m depth during daylight, and from 0 to 50 m at night on the north coast of Honshu (Japan) (Bower and Miyahara 2005). These bathymetric differences are due to the close relationship between depth distribution and the depth of the Deep Scattering Layer (DSL) (Yano et al. 2000). On the other hand, highest catches of the diamond squid arise at 14–15°C and the peak CPUE (Catch Per Unit Effort) occurs in winter period (December-February) and in areas close to upwelling events (Bower and Miyahara 2005).

Considering sightings of egg masses, the existence of captures of *Thysanoteuthis rhombus* in the Canary Islands, although neither the used gear nor the period of fishing are the suitable ones, the oceanographic conditions when highest catches occurred, which are frequent in the Canary archipelago especially during winter periods (December-March), and comparing this information with those of other regions of the world where an industrial fishery has developed on this species, we suggest that Canarian waters are a good candidate for developing a commercial exploitation of this species. The fishing infrastructure of the Canary archipelago would favor the development of this fishery. However, to develop this fishery in the Canary Islands would require the adoption of gear similar to that employed by Japanese fishermen (Aiken et al. 2007). Development of a *Thysanoteuthis rhombus* fishery could diminish the existing pressure on overexploited resources of coastal waters in the Canary archipelago, of which a great number of small fishing communities distributed along their coasts survive (Tuya et al. 2005). A potential Canary Islands *Thysanoteuthis rhombus* fishery would develop in bathyal depths, which are close to the shoreline because of the volcanic origin of the islands, especially in western islands (La Palma, La Gomera, El Hierro and Tenerife).

The possibility for development of a small-scale *Thysanoteuthis rhombus* fishery needs to be assessed in detail by pilot fisheries and scientific and economic studies with following considerations: a) it would be necessary to evaluate this fishery sustainability at short and long term; b) it would be needed to evaluate the potential impacts on the ecosystem, especially on resident populations of marine mammals, such as, Risso’s, bottlenose, and rough-toothed dolphins, short-finned pilot whales, sperm whales, Blainville beaked whale, and Cuvier’s beaked whale. Their populations currently support a profitable tourist activity related to whale watching some islands of the Canary archipelago.
